# Comparative observer effects in 2D and 3D localization tasks

**DOI:** 10.1117/1.JMI.8.4.041206

**Published:** 2021-03-18

**Authors:** Craig K. Abbey, Miguel A. Lago, Miguel P. Eckstein

**Affiliations:** University of California Santa Barbara, Department of Psychological and Brain Sciences, Santa Barbara, United States

**Keywords:** observer templates, classification images, localization tasks, volumetric imaging

## Abstract

**Purpose:** Three-dimensional “volumetric” imaging methods are now a common component of medical imaging across many imaging modalities. Relatively little is known about how human observers localize targets masked by noise and clutter as they scroll through a 3D image and how it compares to a similar task confined to a single 2D slice.

**Approach:** Gaussian random textures were used to represent noisy volumetric medical images. Subjects were able to freely inspect the images, including scrolling through 3D images as part of their search process. A total of eight experimental conditions were evaluated (2D versus 3D images, large versus small targets, power-law versus white noise). We analyze performance in these experiments using task efficiency and the classification image technique.

**Results:** In 3D tasks, median response times were roughly nine times longer than 2D, with larger relative differences for incorrect trials. The efficiency data show a dissociation in which subjects perform with higher statistical efficiency in 2D tasks for large targets and higher efficiency in 3D tasks with small targets. The classification images suggest that a critical mechanism behind this dissociation is an inability to integrate across multiple slices to form a 3D localization response. The central slices of 3D classification images are remarkably similar to the corresponding 2D classification images.

**Conclusions:** 2D and 3D tasks show similar weighting patterns between 2D images and the central slice of 3D images. There is relatively little weighting across slices in the 3D tasks, leading to lower task efficiency with respect to the ideal observer.

## 1 Introduction

Three-dimensional “volumetric” images are widely used in medical imaging for many purposes and across various imaging modalities. Volumetric images are appealing at a fundamental level because the 3D spatial relationships present in the body can be faithfully represented in the image up to the practical limits of contrast, resolution, and noise.^1^^,^^2^ However, even with the development of stereo and holographic display techniques,^3^^–^^5^ 3D images are typically displayed on a 2D monitor, which necessitates some method of accommodating this dimensionality mismatch. Many techniques for image display have been developed, ranging from surface rendering and fly-through approaches, to simultaneous multiview display.^6^^–^^8^ Nonetheless, it is not uncommon for volumetric images to be read in a clinical setting by simply scrolling through a “stack” of 2D sections.

Scrolling replaces one of the spatial dimensions of a 3D image by mapping it into a temporal component where the reader controls the scrolling rate and direction as they search a 3D image for some target of interest. This has many potential consequences. In this work, we are interested in what happens when the target of interest is spread across multiple sections of the 3D image in the presence of masking noise. In principle, the most effective way to find such a target will involve integrating information across these 2D sections. It is of interest at a fundamental level to know how human observers perform such an integration. At a more practical level, it is often the case that task-based psychophysical assessments of image quality in volumetric imaging modalities replace a fully 3D task with a simpler (and faster) 2D task in a single slice (e.g., Refs. 9–12). Here, the question is whether the restriction to a single “slice” image fundamentally changes the way that human subjects perform the task, potentially biasing the results of such studies. The experiments reported here are intended to make contributions to both questions.

Since our motivation is not specific to any particular (3D) imaging modality, our approach is based on generic simulated images. Simulated images have the advantage of being experimentally controllable and well characterized statistically. Both of these qualities are important for the analyses we perform. Image simulations have a long history of use establishing observer effects that impact the fields of medical image perception and vision science. Some examples of this are characterizations of visual efficiency in noise,^13^^–^^15^ observer adaptation to image correlations,^16^^–^^20^ internal noise,^21^^–^^23^ and the effect of different types of tasks.^24^^–^^27^ All of these works have used 2D simulated images to evaluate properties of human observers. There have been far fewer studies comparing and modeling observer effects between 2D and 3D images, with some notable examples^28^^–^^33^ nonetheless, which makes the simulated-image approach more appealing for this purpose.

We investigate integration across multiple 2D sections of a volumetric image using a forced-localization task to evaluate and compare spatial weighting in noise-limited 2D and 3D images, where user-controlled scrolling is used to navigate the through the slices of 3D images. The stimuli are constructed so that an ideal observer (IO) is theoretically and computationally tractable,^27^^,^^34^ which allows us to evaluate localization efficiency as a measure of how much task-relevant information is being accessed by the human observers. The classification-image technique is used to evaluate spatial weighting used by observers to perform the tasks, which shows how information in the images is being accessed. We believe that the approach taken in this work, extending a preliminary conference report,^35^ is a novel application of efficiency and classification images to compare 2D and 3D forced-localization tasks, which build on recent results for 2D localization tasks.^27^^,^^36^ The noisy images we use are generated as Gaussian random fields with either a white-noise texture, as an approximation of acquisition noise, or a power-law texture, as an approximation of anatomical variability.^37^^–^^40^ The targets to be localized are spheres (disks in 2D) of two different sizes that have been filtered and downsampled to approximate the spatial-resolution properties of modern volumetric imaging x-ray CT scanners.^41^^–^^43^

## 2 Methods

This study comprises a total of eight experimental conditions that explore localization performance across three factors: image dimension (2D and 3D), target size (large and small), and noise texture (power-law noise and white noise). Image dimension is the primary focus of the study with target size and noise texture effects giving some sense of robustness of the findings across different kinds of images.

### 2.1 Image Stimuli

All of the images used in this study are simulations generated in 3D. The 2D condition is implemented in the image display code, which only allows viewing of the slice containing the target center. The images are intended to roughly approximate a region of interest in high-resolution computed tomography (CT) imaging, with a nominal isotropic voxel size of $0.5\;{\mathrm{mm}}^{3}$ and a total 3D image size of 256×256×256.

**Fig. 1:**
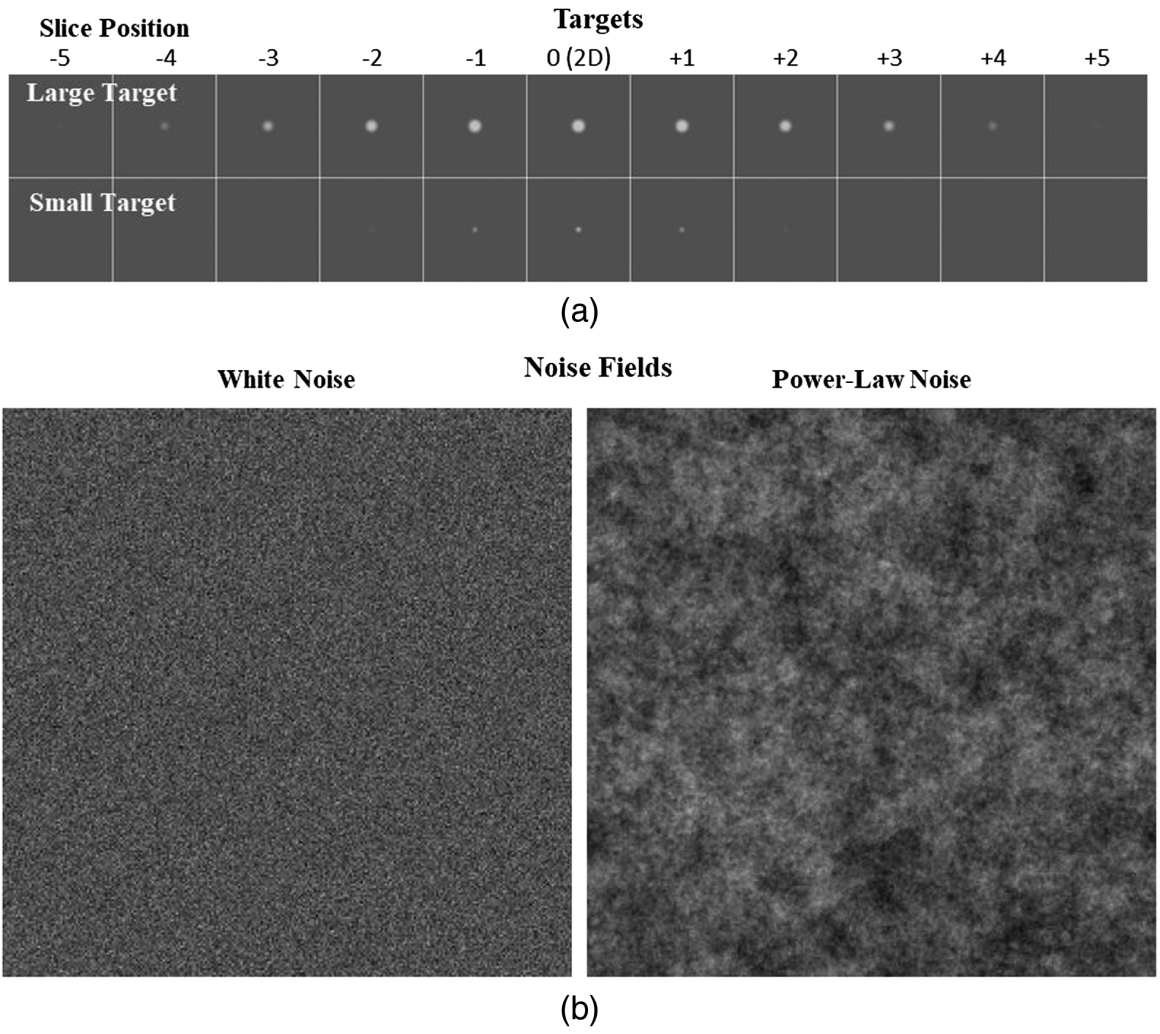
(a) Targets and noise. The 3D profile of the large target (upper row) and small target (lower row) are shown for ±5 slices from the target center. For the 2D task, only the central slice (slice number 0) appears in the image. (b) Examples of the white noise (left) and power-law noise (right) textures used in the experiments. In the 3D tasks, these would be a single slice from a volumetric image.

Figure 1(a) shows the two targets used in these experiments. Both targets are blurred spheres of constant intensity. The “large” target (Lg) has a 4 mm diameter, and the “small” target (Sm) has a 1 mm diameter. The large target extends in the z-direction over five slices in both directions while the small target extends over two slices in both directions. The blurring of the target profiles is intended to roughly approximate a system transfer function in an imaging context. For simplicity, we use a rotationally symmetric blurring function implemented as a filter in the FFT domain. For a radial frequency component defined as $f=\sqrt{f_{x}^{2}+f_{y}^{2}+f_{z}^{2}}$, the transfer function filter is given by a cosine roll-off function from DC (f=0) to Nyquist ($f_{\mathrm{Nyq}}=1.0\;\mathrm{cyc}/\mathrm{mm}$):

$$T(f)=\{\begin{array}{ll} 0.5+0.5\;\cos(\pi f/f_{\mathrm{Nyq}}) & 0\leq f\leq f_{\mathrm{Nyq}}\\ 0 & f>f_{\mathrm{Nyq}} \end{array}.$$ (1)

The transfer function falls off from 1 at f=0 to 0 at $f_{\mathrm{Nyq}}$ with a full-width at half-max at 0.5  cyc/mm, which is roughly consistent with the transfer properties of high-resolution CT scanners.^43^ Note that target amplitudes are defined in this work as the amplitude of the disks before filtering by the transfer function. This makes them analogous to the amplitude of lesions in tissue for the medical-imaging context.

Figure 1(b) shows sample slices for the two Gaussian noise textures used as image backgrounds. The two textures consist of white-noise (WN), in which every voxel is an independent Gaussian process, and a so-called “power-law” noise (PL) in which the power spectrum of the noise fields obeys a power-law, $1/{\left(f+\varepsilon \right)}^{3}$, with a small offset (ε=0.0078  cyc/mm) to avoid instability near f=0. The power spectra of both processes are scaled so that the voxel standard deviation is 20 gray levels, and a mean background of 100 gray levels is used, which keeps in the images mostly well within the 8-bit display range (256 gray levels) of the monitor. Any voxels outside the 8-bit range are truncated to the nearest boundary (0 or 255).

Image backgrounds are generated by initially sampling from a standardized normal random number generator, taking the 3D FFT, multiplying by the square root of the power spectrum, inverse transforming, and then adding the mean background level. A target profile at a specified target amplitude is then added to the image background at a random location in the central 128×128×128 region of the volume, and the result is truncated to the 8-bit gray-level range of the monitor. A set of five target amplitudes are mixed across the trials. The procedure for determining these are described in the next section.

### 2.2 Forced Localization Task

Forced localization is a generalization of the multiple-alternative forced-choice paradigm. The target is always present in the image at an unknown random location, and in each trial the subject identifies the location they believe is most likely to be the target center. The response is considered correct if it falls within a distance of 6 pixels (3.6 mm radius on the display) of the actual target center.

**Fig. 2:**
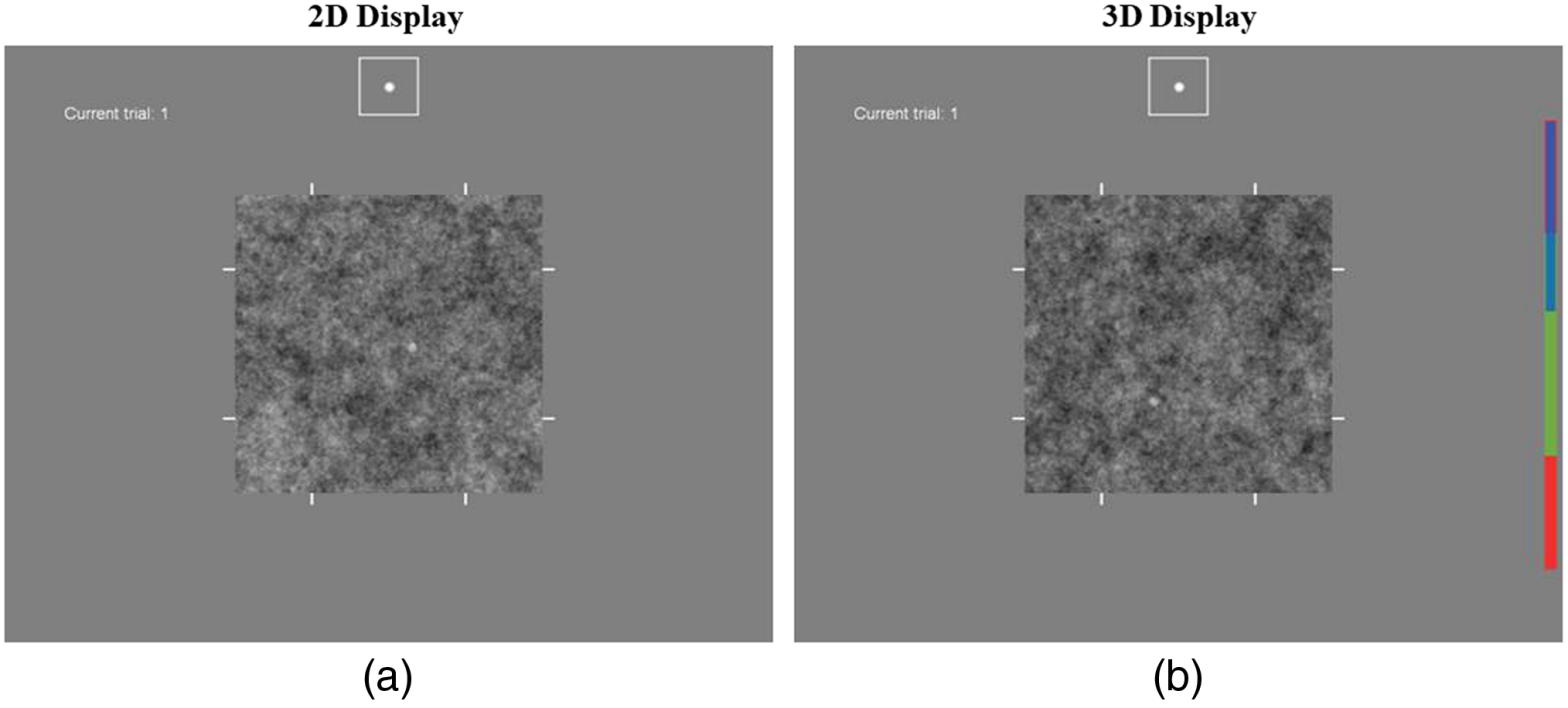
Localization displays. Display windows for (a) the 2D and (b) 3D stimuli are shown. The example images shown are for the large-target in power-law noise condition. A reference image of the noiseless target is displayed at the top of the window (in the box), and the trial number is shown in the upper left side of the window. The small hash marks on the edge of the image indicate the X-Y search region. On the 3D display, the scroll-bar on the right side shows the depth of the current slice in the Z direction (blue) along with the depth range (green) of the 3D search region.

Figure 2 shows the forced-localization interface for the 2D and 3D tasks. For the 2D tasks, a single slice is shown in the interface, as in Fig. 2(a). This slice is selected to pass through the center of the target in the z direction. The observer responds by double-clicking a mouse-driven pointer on the selected location, which must be in the central 128×128 region of the image (i.e., inside the hash marks at the edge of the image). Responses outside of this area are ignored, and a trial lasts until a valid response is obtained.

In the 3D task shown in Fig. 2(bB), the subjects need to navigate through the volume as part of the localization response. This is accomplished using a mouse click-and-drag, up or down through the z range of the 3D image. For fine tuning the slice selection, the up and down arrows on the keyboard can be used to move a single slice at a time. The scroll bar on the right side of the 3D interface is used to indicate the position of the current slice in the 3D stack. It also indicates the middle 128 slices of the z range (in green). Localization responses are only accepted within this range.

In each experimental condition, the performance is assessed in two phases. In the first “training” phase, an adaptive staircase is used to estimate the 80% correct target amplitude. We use a three-down one-up staircase in which three correct responses result in the next trial having a 15% reduced target amplitude and a single incorrect response leading to a 15% increased target amplitude. This staircase is known to oscillate around the 80% correct threshold.^44^ The staircase starts at high amplitude to give the observer the opportunity to get familiar with the task. It typically takes 20 to 30 trials for the first incorrect response to be made. The staircase is run for a total of 12 reversals, in which the amplitude goes from decreasing to increasing or vice-versa. The threshold estimate is derived from the geometric mean of the target amplitude over the last eight reversals. The adaptive staircase procedure is run three times, with the final training threshold estimate being the average of the three runs.

A total of 500 forced localization trials are used for the test set, which uses five different target amplitudes that are randomly mixed throughout the trials (100 trials at each of the amplitudes). This includes the 80% correct threshold estimated from the training runs, as well as ±10% of this threshold and ±20% of this threshold. The range of amplitudes gives us some ability to assess the subjects’ psychometric functions and also ensures that there will be a reasonable frequency of difficult cases leading to a sufficient number of incorrect responses for estimating a classification image. In each trial, the display software records the index of the stimulus, the target amplitude of the trial, the true location of the target, the localization response of the subject, and the reaction time from stimulus display to the recording of a valid mouse click. The true target location is given as x, y, and z indices of the target center. The localization response is coded as x, y, and z indices of the subject-selected image pixel. In the 2D task, the z index of the localization response is constrained to be the target z index. The proportion of correct responses (PC) is used as the measure of performance for a given amplitude. It is computed for each of the five amplitudes tested.

The experimental data were collected using a clinical review monitor (Barco Inc.) calibrated to the DICOM standard over a measured luminance range of 0.04 to $165.7\;\mathrm{cd}/m^{2}$. Images were magnified by a factor of 2 for a displayed pixel size of 0.6 mm, given the native (isotropic) pixel size of 0.3 mm. Subjects were encouraged to position themselves at a comfortable viewing distance, which was typically between 50 and 100 cm from the monitor face. For a subject at the center of this range, 21.8 pixels subtend a visual angle of 1 deg.

A total of five subjects conducted the studies reported here under an IRB-approved human subjects protocol at the authors’ institution. The four 2D experiments were completed in roughly 30 to 45 min per condition, but the 3D experiments took considerably longer, requiring 3 to 4 h for each condition. The total time to complete the study for each subject was roughly 20 h, spread over multiple sessions at the workstation. Four of the subjects were naïve to the purpose of the research and compensated for their time, the other subject is the first author.

### 2.3 Ideal Observer

The Ideal Observer, described in a previous publication,^27^ was used in the computation of efficiency. We briefly review the computations involved in evaluating the IO on a given image here. The first step involves a convolution with the prewhitened matched filter,^45^ then exponentiation of the result (within the search region) to form a posterior distribution on target location. A second scanning operation with a 6-pixel radius disk (in 2D) or sphere (in 3D) is used to compute the posterior utility of each point in the search region. The point that maximizes this utility function over all possible locations is the IO response for the trial.

**Fig. 3:**
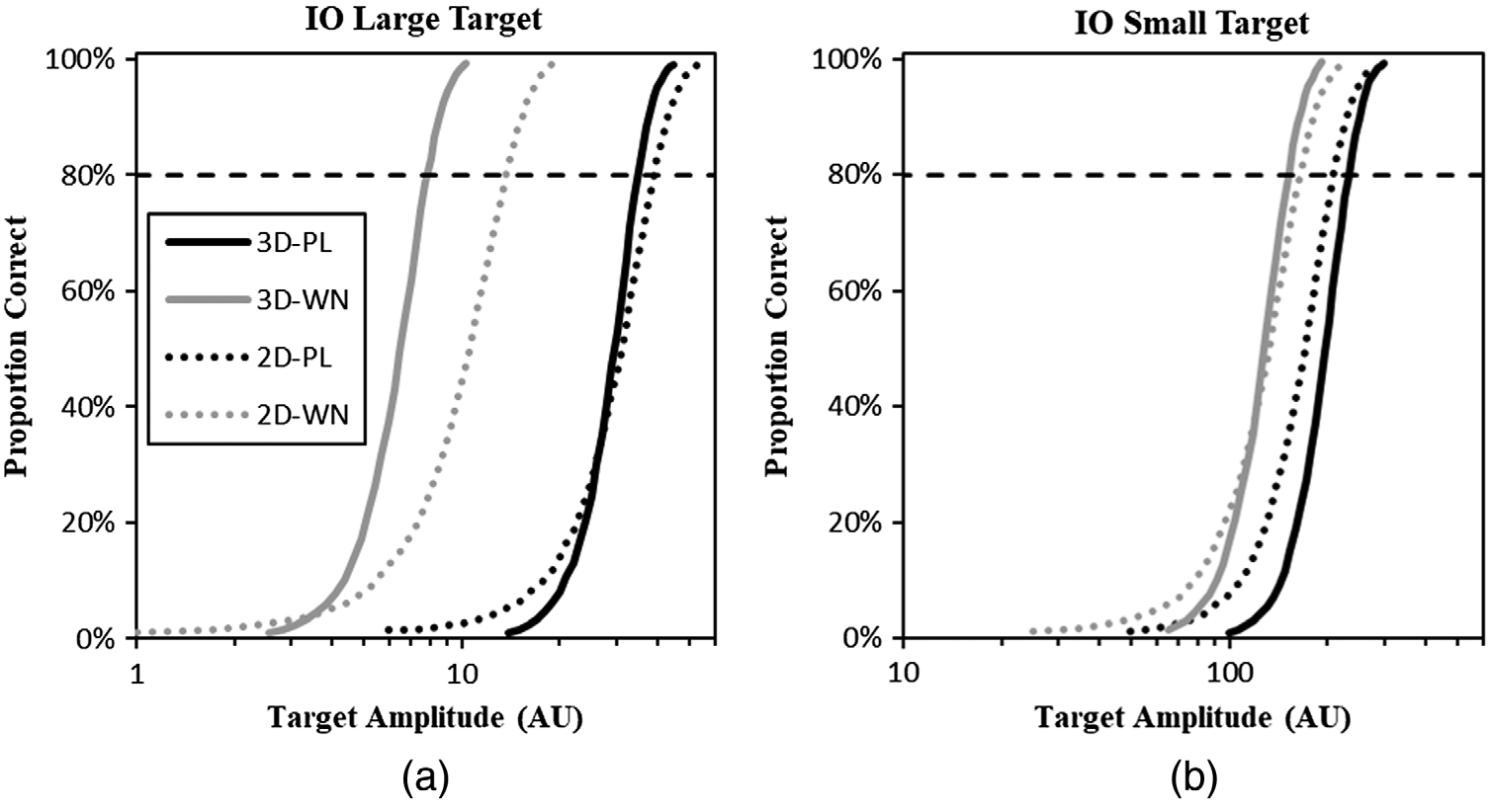
IO psychometric functions. IO psychometric functions are shown for the tasks with (a) the large target and (b) small target. Each plot shows 3D and 2D performance in power-law noise (PL) and white noise (WN). Each point in the plot is the outcome of 5000 Monte-Carlo trials. The targeted performance level of 80% in indicated by the dashed line. Note the different ranges of the logarithmic x axis showing much lower large-target thresholds. Legend applies to both plots.

Monte-Carlo studies using many independent sample images at a given target amplitude are used to assess the performance of the IO in terms of the proportion of correct localizations (PC). Evaluations at a range of target amplitudes can be used to obtain the ideal-observer psychometric function, which shows how target amplitude affects performance in each condition. Ideal-observer psychometric functions in all eight experimental conditions are plotted in Fig. 3 using 5000 Monte-Carlo trials at each of the target amplitudes. These data are used to get ideal-observer amplitude thresholds for the efficiency computations described next.

### 2.4 Amplitude Thresholds and Efficiency

**Fig. 4:**
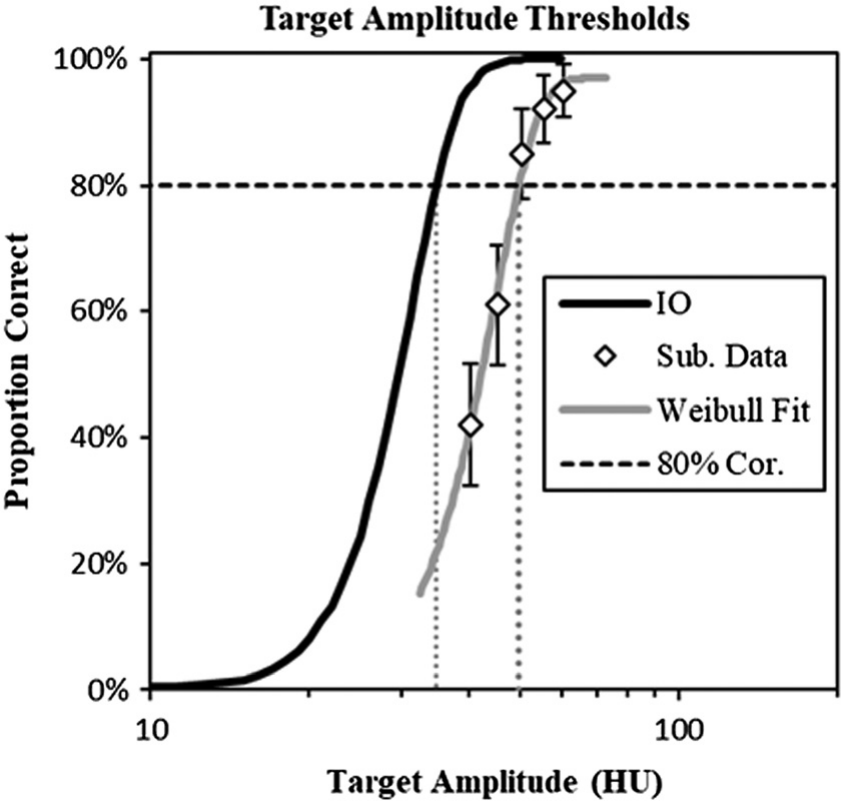
Target amplitude threshold computation. The plot shows how the 80% correct target threshold and task efficiency are computed for a set of observer performance data. The threshold amplitude ($A_{\text{Sub}}$) is derived from a Weibull psychometric curve that is fit to psychometric data. An equivalent threshold for the IO ($A_{\mathrm{IO}}$) is determined from the Monte-Carlo performance evaluations. Efficiency is defined as the squared ratio of these two target amplitudes.

Figure 4 shows how subject data and an ideal-observer psychometric function are used to obtain an estimate of human-observer efficiency for a given experimental condition. As described above, the psychophysical experiments evaluate five different target amplitudes in each condition from which five performance levels are estimated for each subject. These points are used to fit a Weibull psychometric function,^46^^,^^47^ PC(A), defined as

$$PC(A)=P_{B}+(1-P_{B}-P_{E})(1-2^{-{\left(\frac{A}{\lambda }\right)}^{k}}),$$ (2)

where $P_{B}$ is the baseline probability of a correct response (0.34% in 2D and 0.04% in 3D), $P_{E}$ is the lapse rate (assumed to be 3%), λ is the half-rise amplitude, and k controls the steepness of the psychometric function. The λ and k parameters are fit using maximum likelihood, assuming observed subject PCs represent binomial proportions. Once the psychometric function has been determined, the 80% correct amplitude threshold is computed by setting PC(A)=0.8 in Eq. (2), and solving for A. This is seen in Fig. 4 as a vertical line from the intersection of the 80% correct line with the Weibull psychometric function to the x axis, defining the subject’s amplitude threshold, $A_{\text{Sub}}$.

The 80% correct amplitude threshold for the IO is computed by a similar process from the IO psychometric data described above. Since these data are generated from many more trials than the human data (5000 trials per datum instead of 100), and a much finer sampling of amplitudes (50 instead of 5), the IO threshold is found by linear interpolation between the nearest two points, yielding $A_{\mathrm{IO}}$. Efficiency with respect to the IO is then defined as the ratio^48^^–^^50^

$$\eta ={\left(\frac{A_{\mathrm{IO}}}{A_{\text{Sub}}}\right)}^{2}.$$ (3)

### 2.5 Classification Images

Classification image analysis follows the technique described previously for forced-localization tasks.^27^ The classification images are estimated from noise fields of the image stimuli in incorrect trials.^51^^,^^52^ Within each condition and within each subject, these noise fields are all aligned to the (incorrect) response location and then filtered with the inverse-covariance matrix to disambiguate the effects of noise correlations. Since the images are generated from a stationary Gaussian process, this step is implemented through finite Fourier transforms and the inverse noise power spectrum. The resulting filtered noise fields are then averaged to obtain the raw classification image for each subject in each condition. For the 3D images, this process is implemented using the full 3D noise field and 3D inverse-covariance filtering. In the 2D conditions, we use the noise field of the displayed 2D slice. In this case, inverse-covariance filtering is implemented using the slice power-spectrum, which is derived from the 3D power spectrum by integrating in z. The resulting classification images are averaged across subjects for evaluating group effects of the experimental conditions.

The raw classification images can be quite noisy themselves, particularly in the power-law noise condition where low power-spectral density at higher frequencies can amplify estimation error. We use two methods to control for noise: smoothing and spatial windowing. The smoothing operation is implemented by filtering in the 2D frequency domain, with $f_{2D}=\sqrt{f_{x}^{2}+f_{y}^{2}}$. For 3D classification images, smoothing is applied to each slice independently. We apply smoothing filters that are unity for $f_{2D}<0.5\;\mathrm{cyc}/\mathrm{mm}$, and roll off for $0.5\leq f_{2D}\leq 1.0\;\mathrm{cyc}/\mathrm{mm}$ with a cosine-bell profile.

### 2.6 Scanning Models

Classification images are most readily interpreted as representing an estimate of the weights of a linear template model. This has been demonstrated analytically for detection tasks at a fixed location^53^^–^^55^ and empirically for tasks that involve search such as the forced-localization tasks used here.^27^^,^^56^ In localization tasks, the linear template is assumed to scan the entire search region by a convolution operation, much like the first step of the IO model described above. The localization response of the model is typically generated by taking the maximum response of the template within the search region.

When a classification image is used as the linear kernel of a scanning model, the estimation error in the classification image can bias performance of the model. Since estimation error is unlikely to be well tuned to a target profile, this bias is typically toward lower performance. To minimize this effect, we implement a number of steps to control noise in the classification images, including frequency filtering, spatial windowing, and radial averaging. These are described in Sec. 4.3.

## 3 Results

The primary analyses of the experiments are presented here, averaged over subjects. These include the observed amplitude thresholds and efficiency, response times, and classification images in each of the experimental conditions.

### 3.1 Task Performance

**Fig. 5:**
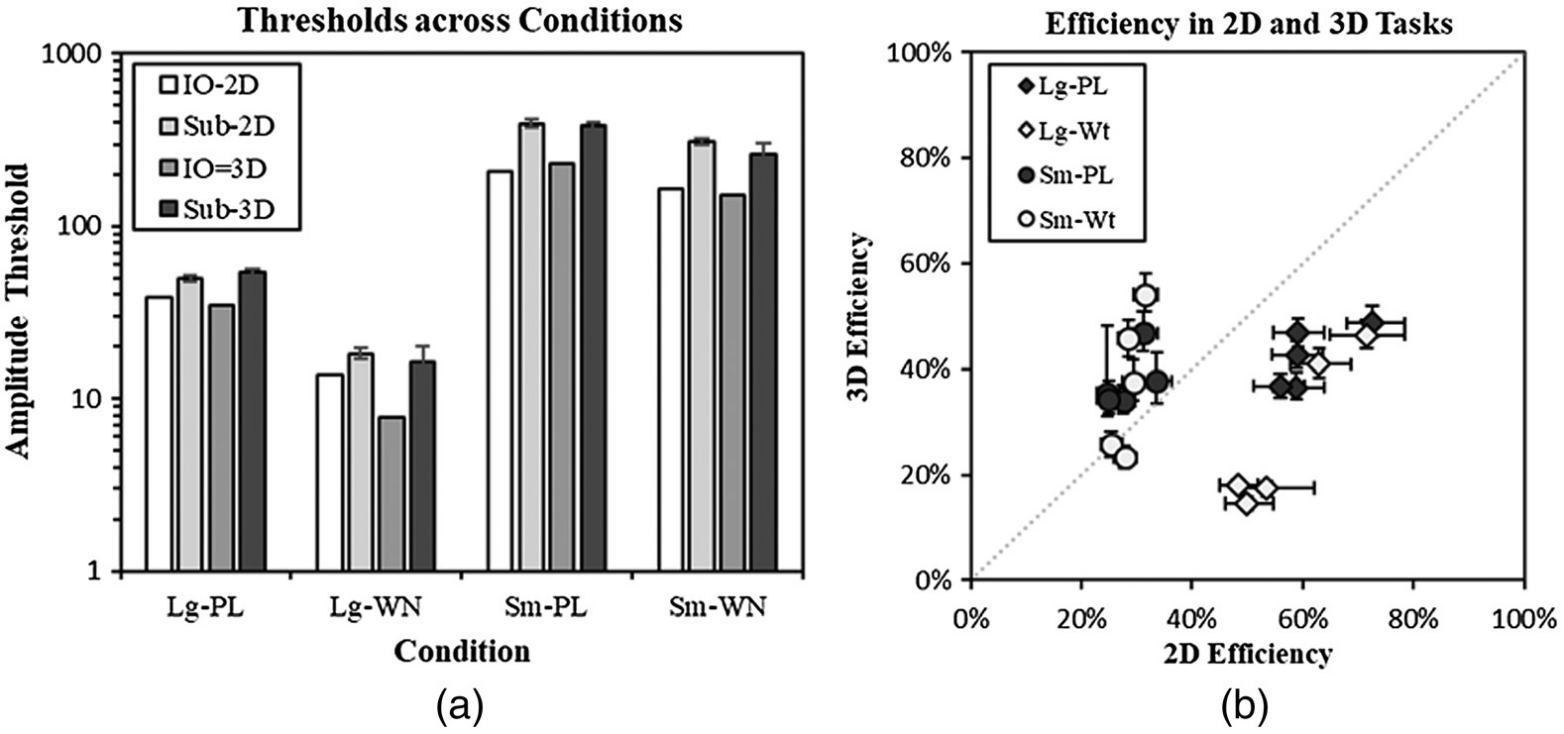
Amplitude thresholds and task efficiency. (a) Thresholds for each condition (Lg, large target; Sm, small target; PL, power-law noise; WN, white noise) are plotted for the IO and the average across human subjects (error bars represent a 95% confidence interval). (b) Subject efficiency is plotted as a scatterplot comparing 2D and 3D search conditions for each of the five subjects. The error bars are 95% confidence intervals generated by bootstrapping across sessions.

Figure 5 summarizes estimated amplitude thresholds for both the IO and the subjects, as well as statistical efficiency of the subjects according to Eq. (3). The amplitude thresholds in Fig. 5(a) vary considerably across the different target-size and noise-texture conditions but are relatively consistent across 2D and 3D display conditions. On average, the relative difference between subject amplitude thresholds in 2D and 3D tasks is 10.5% (min: 3.7%; max: 19.3%), and the qualitative effect of differences in target size and/or noise texture are identical. The IO thresholds are also qualitatively consistent across target size and noise texture conditions, even though the large-target white-noise condition has a 2D threshold that is 75% higher than the 3D condition.

The scatterplot of subject efficiency in Fig. 5(b) shows a clear dissociation between large targets, which are more efficiently localized in 2D, and small targets, which appear to be more efficiently localized in 3D. These differences are statistically significant (paired comparison t-test across subjects) in all cases except for the small-target white-noise condition. The three significant differences all survive a false-discovery rate (FDR) correction for multiple comparisons at the 5% level.^57^ The FDR-corrected p-values are Lg-PL p<0.013; Lg-WN p<0.0016; Sm-PL p<0.019; Sm-WN p<0.156. We will return to this dissociation in Sec. 4.

### 3.2 Response Times

Table 1 shows the response times in each condition, computed as the median response time averaged across subjects (± the standard deviation across subjects). Response times are given for all trials and then broken into trials in which the subjects responded correctly or trials in which the subjects responded incorrectly. Across target-size and noise-texture conditions, 3D trials take 8.9 times longer on average than 2D trials to generate a localization response. This is not surprising given the additional time needed to scroll through the search volume in 3D localization trials. Nonetheless, this larger response time difference does illustrate a substantial practical difficulty of investigating 3D image tasks.

**Table 1:** Median response times.

	Dim	Lg-PL	Lg-WN	Sm-PL	Sm-WN
All trials	2D	2.1±0.6	1.9±0.05	2.2±0.6	1.9±0.5
	3D	16.5±2.5	17.4±5.0	21.5±3.7	17.4±2.6
Correct trials	2D	2.0±0.5	1.8±0.4	2.1±0.6	1.8±0.5
	3D	13.8±2.3	15.1±3.9	19.3±3.3	15.1±1.9
Incorrect trials	2D	3.6±2.0	2.7±1.2	3.4±1.8	2.6±1.0
	3D	40.3±16.7	38.5±21.2	56±21.8	34±8.9

Compared to median times for all trials, correct trials are generally somewhat faster and incorrect trials are generally substantially slower. In 2D tasks, correct trials are 5.8% faster on average and incorrect trials are 51% slower. In 3D tasks, correct trials are generally 13% faster and incorrect trials are 132% slower. It is clear that when subjects make an incorrect localization response, they have spent a relatively large amount of time searching for the target, particularly in the 3D tasks.

### 3.3 Classification Images

**Fig. 6:**
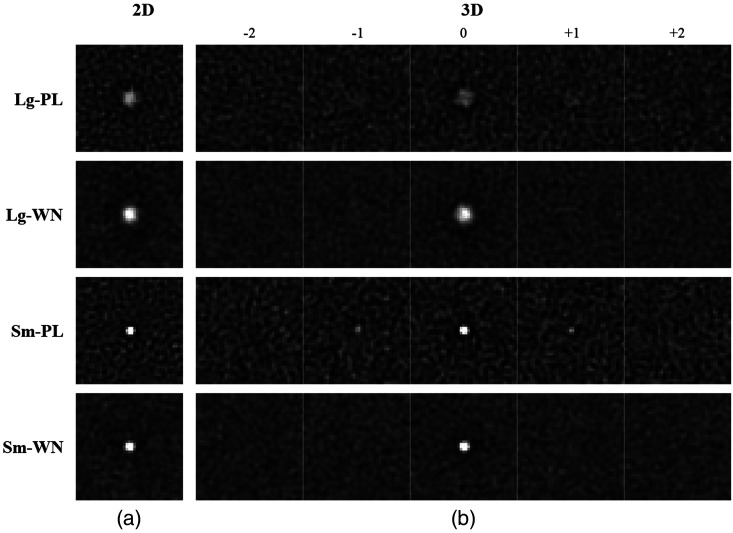
Classification images. The average classification image across subjects is shown for each condition in (a) the 2D and the central five slices of (b) the 3D tasks (response slice −2 to response slice +2). Smoothing filters have been applied to the images.

The average classification images, estimated as described in Sec. 2.5, are shown in Fig. 6. The left column of the panel in Fig. 6(a) is the 2D classification images for each target-size and noise-texture condition. The remaining portion of the panel shows the central five slices of the 3D classification images. The classification images have been frequency filtered for noise control according to the methodology described above (1 to 0.5  cyc/mm and rolled off to zero at 1  cyc/mm).

In the 2D portion of the panel, the classification images all have a center-surround profile, where a bright central region of positive weights are surrounded by a darker region of negative weights. The classification images are clearly tuned to the size of the target (i.e., larger areas of activation for larger targets). The width and magnitude of the surround appears to vary across conditions. The central slice of each 3D classification image is very similar in appearance to the 2D classification image. Off of the central slice, the activation appears to be much weaker, if it can be seen at all. There is some evidence of weak positive activation at ±1 slice. But given that the small signal extends over a total of five slices, and the larger target extends over 11 slices, this represents very limited use of multiple slices.

## 4 Discussion

### 4.1 Comparisons with Prior Investigations

The results of our studies can be related to findings in some earlier studies. Reiser and Nishikawa^30^ compared 2D and 3D images in a free search task with noise structures that are very similar to what is used here (white noise and power-law noise) and targets that are closer in size to the large target in this work. They found a pronounced improvement in performance for 3D images in the white noise backgrounds, and little—if any—improvement for the power law noise. Balta et al.^32^ also used a power-law background (with additional orientation parameters) with blurred disk targets in a signal-known-exactly task. In this case, a more realistic image formation model was used that modeled the limited angular range of digital breast tomosynthesis. They also found similar performance between 2D and 3D images, consistent with Reiser and Nishikawa.

We find similar results in Fig. 5(a) for the ideal- and human-observer amplitude threshold data, although our difference is somewhat less dramatic than the finding in Reiser and Nishikawa. In white noise, the large-target amplitude thresholds drop in 3D relative to 2D, whereas in power-law noise they stay approximately the same. Thus, the absolute performance effects appear to have some robustness properties. However, Fig. 5(b) shows the importance of considering task efficiency as well. While observer performance localizing the large target is roughly equivalent in 3D and 2D images (the 3D amplitude threshold is 7% larger for power-law noise and 11% smaller for white noise), the subjects are considerably more efficient in the 2D task than the 3D task (44% more efficient in power-law noise and 108% more efficient for white noise).

### 4.2 Dissociation between Large and Small Target Efficiency

If we consider these tasks from the perspective of the threshold amplitude, shown in Fig. 5(a), then it is clear that the small targets are substantially more difficult to localize accurately than the large targets in both 2D and 3D tasks with thresholds that are 7 to 17 times larger. There are two possible reasons for this large discrepancy: (1) the tasks with small targets are inherently more difficult or (2) human observers are less effective at localizing the small targets. The efficiency values in Fig. 5(b) help disambiguate these two effects by correcting for task difficulty and therefore isolating reader performance effects. In this context, the reader results show a dissociation in which large targets are more efficiently localized in the 2D tasks and the small targets are more efficiently localized in the 3D tasks.

This finding would appear to be at odds with recent studies by Lago, Eckstein, and colleagues,^33^^,^^58^^–^^60^ demonstrating substantial performance reductions for small targets in 3D search tasks. However, it is important to note a fundamental difference between those experiments and the results reported here. Their investigations examine the role of peripheral vision in modulating search performance in 2D and 3D images. Their images can occupy a much larger portion of the visual field than these studies (up to 30-deg visual angle). The search region used in these experiments can be mostly or entirely covered by central vision. Clinical ophthalmology texts define the fovea (including the perifovea) as occupying the central 8 deg of the visual field.^61^ With this definition and our display procedure described, the entire 128×128 search region fits in the fovea at a viewing distance of 76 cm or more. At a close viewing distance of 50 cm, 67% of the search region is covered by the fovea. Given the search region size and subject viewing distance, it is perhaps not surprising that we do not see evidence of peripheral vision effects.

**Fig. 7:**
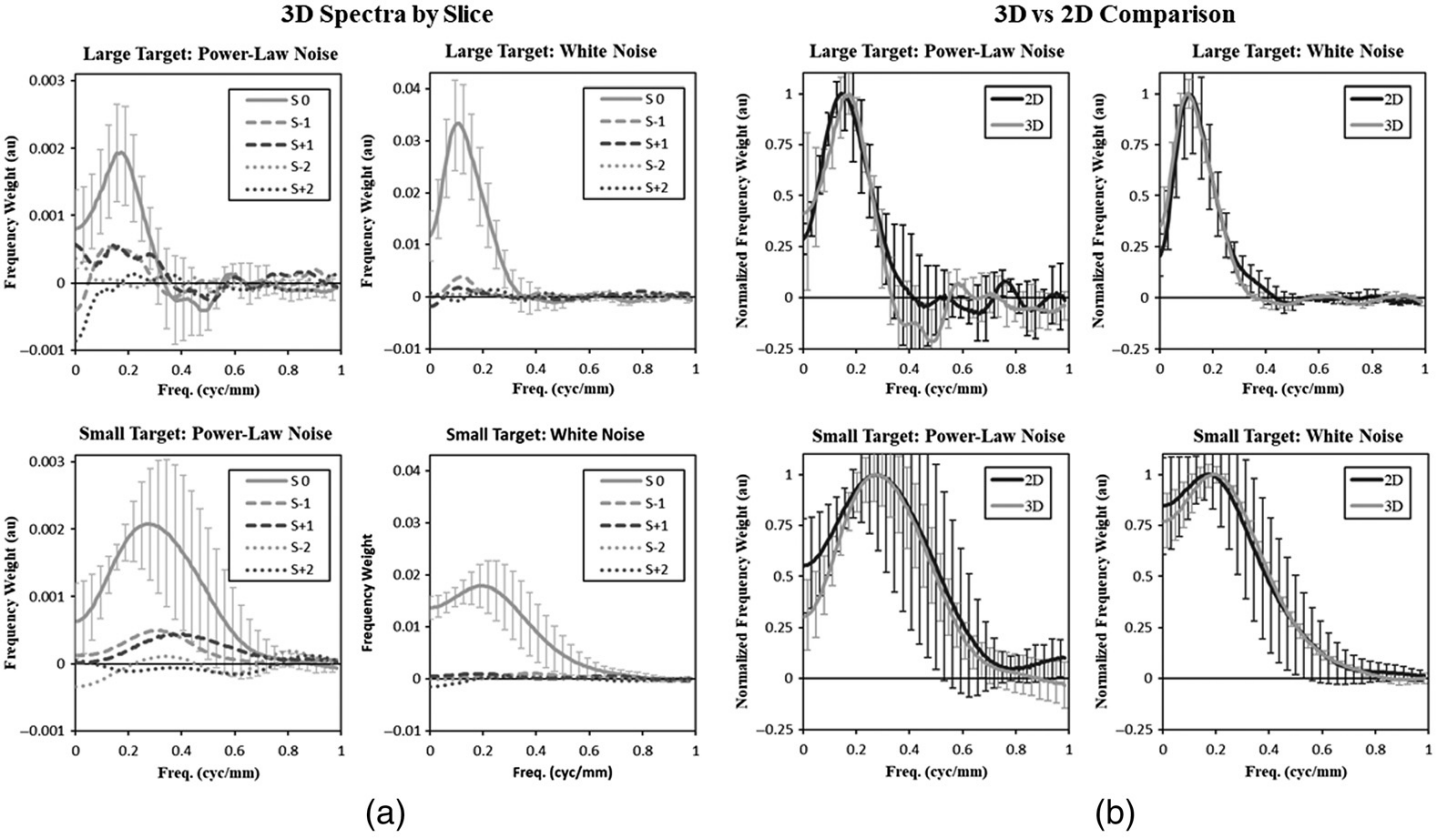
Classification-image spectra. Spectral plots of the classification images are shown. (a) For the 3D classification images, a spectral plot from five slices is shown (−2 to +2 slices from response slice). Error bars representing a 95% confidence interval across subjects are plotted on the central slice (at every fourth point). Error bars on the other slices are similar in magnitude, but not shown for clarity. (b) For the comparison across 2D and 3D classification images, spectral plots from the central slice of the 3D classification image is compared to the 2D spectra after normalization so the peak frequency is 1. The 95% confidence error bars are plotted every fourth sample here as well, with an offset of two samples between the two plots for clarity.

The classification images, on the other hand, suggest that a major source of inefficiency for large targets is the lack of spatial integration across multiple slices in the 3D images, when viewed by scrolling. The spatial weights in the classification images are largely gone after the central slice. This can be seen in the off-center slices of the 3D classification images in Fig. 6. Figure 7(a) shows the classification images in the frequency domain as the average spectral weight at each radial frequency. This gives a more quantitative comparison of the difference between the central slice and the adjacent slices. In both of these figures, there is some evidence for mild weighting of slices immediately adjacent to the central slice in the power-law noise conditions and almost no evidence for off-center weighting in the white noise condition. A failure to integrate target information across multiple slices has a greater effect on efficiency for larger targets that are spread over more slices, consistent with the efficiency results we find. This is also broadly consistent with the use of multiple views for volumetric images in the clinical context, where different views would be used to ensure 3D information is integrated into a final decision.

### 4.3 Similarity between 2D and 3D Classification Images

The 2D classification image is visually similar to the central slice of the 3D classification image, as seen in Fig. 6. Figure 7(b) shows that the average spectral weights are similar as well, with both 2D and 3D classification images adopting bandpass profiles. Table 2 quantifies these similarities in terms of the common bandpass features of peak frequency and fractional bandwidth (FWHM relative to peak frequency). The average relative difference between 2D and 3D conditions is −4% for peak frequency and 8% for fractional bandwidth. For comparison, consider the average relative difference between power-law and white noise, which is −34% for peak frequency and 31% for fractional bandwidth. Alternatively, the average relative difference between the large target and the small targets is 75% for peak frequency and 30% for fractional bandwidth. Thus, relative to other effects in these data, differences between 2D and the central slice of the 3D classification images are small.

**Table 2:** Peak frequency and fractional bandwidth for each condition.

Cond	2D	3D	2D (%)	3D (%)
Lg-PL	0.15	0.17	137	118
Lg-WN	0.11	0.10	150	177
Sm-PL	0.28	0.27	181	146
Sm-WN	0.17	0.20	223	204

This similarity between 2D and 3D classification images, along with the lack of substantive off-center weighting in the 3D classification images, establishes a mechanistic similarity between the 2D and 3D localization tasks. Despite the differences in image display and regardless of the search procedure used, subjects appear to be localizing targets in the 3D images as if they were looking mainly at that 2D slice. This lends some credence to the practice of evaluating 3D images using a single 2D slice, although there are many potential caveats and limitations to this statement as described below.

### 4.4 Classification Images as Kernels of a Scanning Localization Model

The classification image can be interpreted as an estimate of the filter kernel^27^^,^^36^ in the context of scanning models of localization performance. In fact, validation of classification-image estimation for localization tasks is based on generating responses from a scanning linear model and showing that the classification image accurately estimates the kernel of this model. This class of model has been used to understand search in medical images previously,^62^^–^^65^ although the recent results of Lago et al.^59^^,^^60^ serve as a caution when peripheral vision effects may be present. Nonetheless, the classification images can be used to understand how much of the subject’s efficiency is due to the spatial weighting implemented in the scanning kernel and how much is due to other processes in the localization tasks (e.g., inefficient search or internal noise).

Estimation error is an important issue for implementing the classification images in scanning models. Noise in the classification image estimate will tend to reduce performance (and therefore the localization efficiency) of the model since it is unlikely that estimation error will be well tuned to a target profile. To mitigate the effects of estimation error, we use relatively aggressive filtering of the classification images based on the frequency profiles shown in Fig. 7. For the large targets, the smoothing filter extends to 0.3  cyc/mm before rolling off to zero with a cosine profile at 0.6  cyc/mm. For the small targets (which extend further into the frequency domain), the smoothing filter is constant to a frequency of 0.6  cyc/mm and rolls off to zero at 1  cyc/mm (which is identical to the filtering used in Fig. 6). In addition, radial averaging is used to smooth radial bands in the spatial domain, under the assumption of approximate rotational symmetry, and a spatial window is applied under the assumption of a relatively compact filter kernel. This spatial window is also tuned to the size of the targets. For the large targets, the spatial window is constant out to a radius of 4 mm and rolls off to zero at 6 mm with a cosine profile. For the small targets, the spatial window is constant out to a radius of 2 mm and rolls off to zero at 4 mm.

**Fig. 8:**
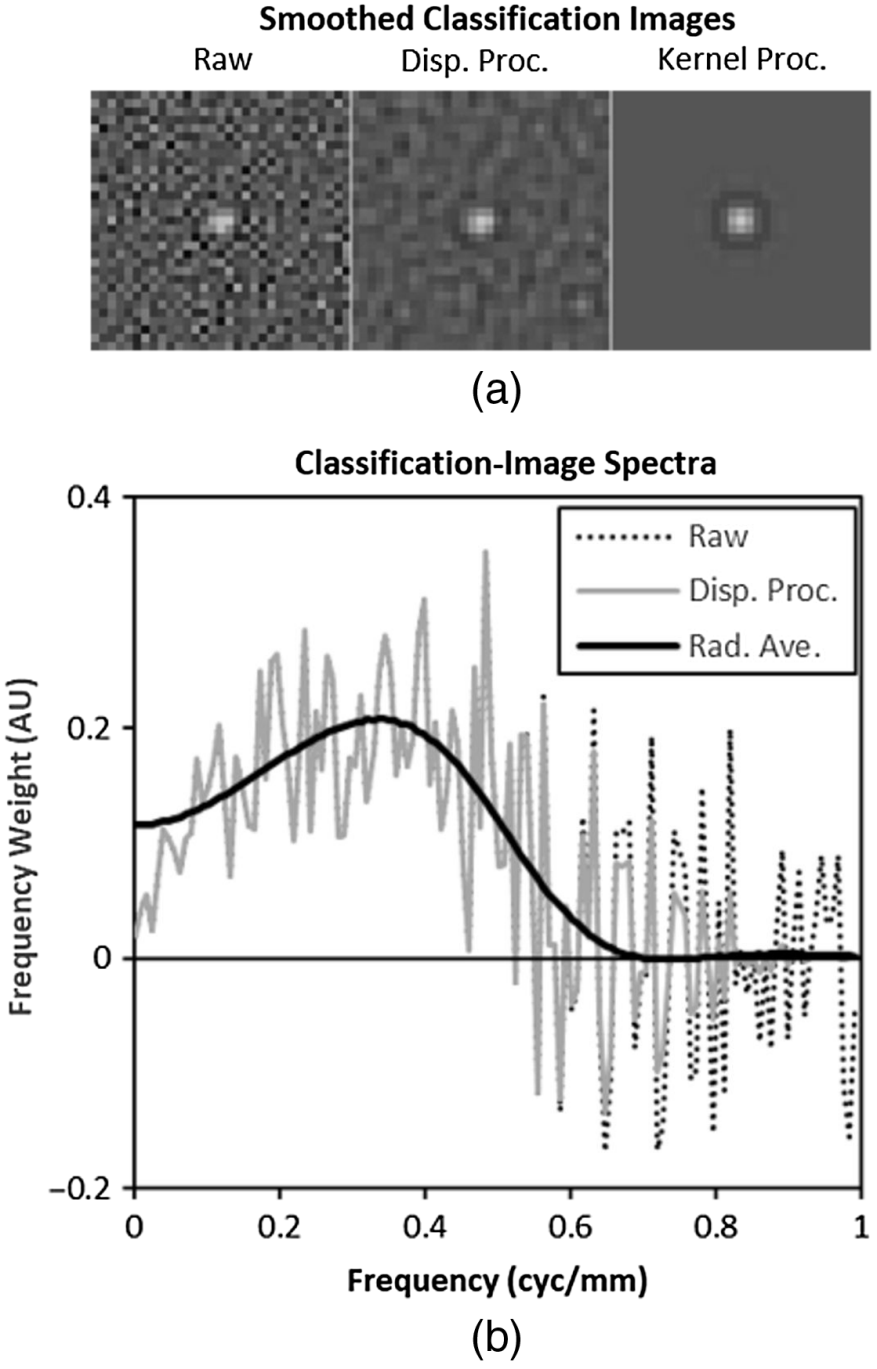
Filtering classification images for scanning models. (a) The images and (b) frequency plots show the effect of smoothing approaches applied to the raw classification image for extracting a scanning kernel. Smoothing for display (Disp. Proc.) as in Fig. 6 is seen to remove some noise, especially at higher spatial frequencies, but not as much as “kernel processing,” which also includes radial averaging and a spatial window (see text). The spectral content of the various smoothed images is relatively similar up to no noise effects.

Figure 8(a) shows an example of the effects of different filtering procedures on the classification image. A raw classification image for a given subject in one of the tasks (PL-Sm) is shown along with the “display processed” version that has been frequency-filtered as in Fig. 6, and a “kernel processed” version that has been processed as described above. The kernel processed image is seen to be largely devoid of visible estimation error. For the 3D classification images, kernel processing is applied to the central three slices, with slices outside this range set to zero. Figure 8(b) shows the real component of the frequency spectrum for the various versions of the classification image. The display processed classification image is seen to have frequencies modulated starting at 0.5  cyc/mm and completely eliminated at 1  cyc/mm, consistent with the filter used to smooth the image data. The spectrum of the kernel-processed classification image is seen to have a spectrum that is generally consistent with the others, but substantially smoother.

**Fig. 9:**
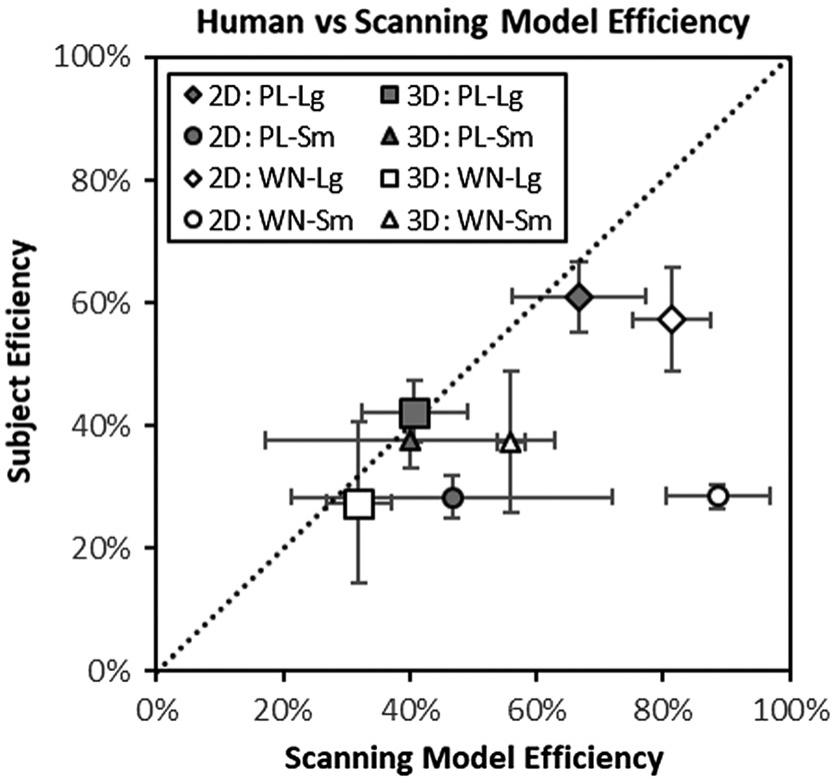
The average efficiency of scanning models derived from classification images is plotted against the efficiency of subjects in each of the eight tasks. Error bars represent 95% confidence intervals across subjects.

Figure 9 shows the average subject efficiency as a function of the average efficiency of the classification-image-derived scanning models. In previous work,^36^ task efficiency has been reasonably well modeled as kernel efficiency minus 12.6% points with a coefficient of determination ($R^{2}$) of 0.86. While that relationship seems to hold reasonably well on average in this data (average kernel efficiency minus average task efficiency is 16.5% points), the association is much weaker with $R^{2}=0.14$. However, one of the eight data points on the plot appears to be driving the lack of association. This point represents the 2D task with a small target and white noise (task efficiency is 28.5% and kernel efficiency is 88.6%). If we exclude this data point, association improves considerably with $R^{2}=0.68$.

This extreme point bears further consideration. The difference between kernel efficiency and task efficiency is more than 50% points. This suggests a relatively optimal kernel combined with substantial deficiencies in other components of task performance, such as incomplete search or internal noise. The task efficiency is relatively low compared to previous studies^36^ that included target localization in white noise, where task efficiency was closer to 60%. It should be noted that the values reported for this condition are relatively consistent across the five subjects, ranging from 25.3% to 31.6%, so the observed value is not driven by a single outlying subject. Thus, it would seem that there may be some aspect of the stimuli or display that leads the subjects to have particularly poor performance despite an efficient kernel in this condition.

### 4.5 Limitations

The discussion above of the extreme point in Fig. 8 indicates that there are some limitations on the interpretation of the specific conditions in this study, particularly in regards to the scanning linear kernel model. It is also important to recognize a few more general limitations in these experiments. The fact that we find little evidence of integration across multiple slices of a 3D image is likely due, at least in part, to the display procedure, which only allows the reader to view the 3D images in a scrolling fashion. This choice has been made deliberately to explore the 3D classification images and see if subjects are capable of integrating multiple slices into a localization response. The result should not be interpreted as a general finding in all 3D image displays.

The images used here are based on Gaussian textures, as needed for computation of localization efficiency and the classification image technique. These images have some general similarity to anatomical variability and acquisition noise, but there are considerable differences as well including differences from smoothing filters and the ramp-spectrum of noise in tomographic imaging modalities. It may be that the results here are specific to such textures and do not extend to more realistic medical images. For example, it is possible that when image structure is present in the image, in the form of patient anatomy, it allows clinical readers to integrate across multiple slices in a way that they do not in these image stimuli. While we recognize these limitations, we also believe that this study presents baseline results that will be useful for understanding human observer performance in 3D images.

## 5 Conclusions

The main finding of this study is the limited and inefficient weighting of multiple slices in the 3D localization tasks, and the similarity of the weighting profile of the central slice to the weighting profile of the 2D tasks. The lack of integration across multiple slices provides an explanation for an observed dissociation in which large targets are more efficiently localized in the 2D tasks, and small targets are more efficiently localized in 3D tasks. This finding is consistent with the common practice of using multiple views of 3D medical images in clinical settings. The similarity between the 2D classification image and the central slice of the 3D classification image provides a rationale for using 2D tasks as a proxy for more time-consuming 3D tasks, but only under the strong assumption that other components of the search process do not disrupt this relationship.

When the observed classification images are used as a simple scanning model of localization performance, the average efficiency of the classification images is ∼10% to 16% greater than the efficiency of the human subjects, which is remarkably consistent with previous findings.^36^ However, this relationship is much weaker than previously reported ($R^{2}=14\%$ or $R^{2}=68\%$ with one outlier excluded), which indicates that other factors in the human subjects or the experimental design impact task performance.
